# Association between prenatal exposure to maternal metal and trace elements and *Streptococcus* infection: A prospective birth cohort in the Japan Environment and Children’s Study

**DOI:** 10.1371/journal.pone.0319356

**Published:** 2025-02-27

**Authors:** Hiroyoshi Iwata, Atsuko Ikeda, Mariko Itoh, Rahel Mesfin Ketema, Naomi Tamura, Takeshi Yamaguchi, Keiko Yamazaki, Rieko Yamamoto, Maki Tojo, Yu Ait Bamai, Yasuaki Saijo, Yoshiya Ito, Reiko Kishi, the Japan Environment and Children’s Study (JECS) Group

**Affiliations:** 1 Center for Environmental and Health Sciences, Hokkaido University, Kita-ku, Sapporo, Japan; 2 Faculty of Health Sciences, Hokkaido University, Kita-ku, Sapporo, Japan; 3 Department of Social Medicine, Asahikawa Medical University, Asahikawa, Japan; 4 Faculty of Nursing, Japanese Red Cross Hokkaido College of Nursing, Kitami, Japan; King Faisal Specialist Hospital and Research Center, SAUDI ARABIA

## Abstract

**Background:**

*Streptococcus* infection is a common and potentially severe bacterial infection which remains a global public health challenge, underscoring the necessity of investigating potential risk factors.

**Aims:**

The present study aims to assess the association between metal and trace element exposure and *Streptococcus* infection using a prospective nationwide birth cohort, the Japan Environment and Children’s Study (JECS).

**Methods:**

The JECS obtained data from over 100,000 pregnancies through 15 Regional Centres across Japan. We assessed toxic metal and trace element levels among pregnant mothers and *Streptococcus* infection among their children, born between 2011 and 2014, at age three to four. Analysis was performed using univariable and multivariable logistic regressions, as well as Quantile g-computation. We also conducted quartile regressions to assess the effects of higher serum selenium levels and potential interactions between selenium and mercury.

**Results:**

Among 74,434 infants and their mothers, univariable and multivariable regression analyses found that selenium and mercury each had an inverse association with *Streptococcus* infection incidence. Quantile g-computation analysis yielded results consistent with the primary regression analyses. Quartile regression suggested that serum selenium levels above the third quartile were inversely associated with later *Streptococcus* infection incidence, but no interaction between selenium and mercury was found.

**Conclusions:**

These findings imply that maternal selenium exposure may have protective effects on *Streptococcus* infection among children. Further studies should explore the role of pediatric selenium in immune responses to infectious diseases, especially *Streptococcus* infection.

## 1. Background

*Streptococcus* infection is the most common pediatric infection. Pediatric *Streptococcus* infection involves a variety of potentially severe clinical presentations, including cellulitis, impetigo, endocarditis, and streptococcal toxic shock syndrome. Among the various types of *Streptococcus* infections, Group A β-hemolytic *Streptococcus pyogenes* (GAS) infection is a common, potentially serious disease among children, which is responsible for about one third of pharyngitis cases in this population [1]. GAS causes over 18 million infections and 500,000 deaths each year [2]. While prompt diagnosis and antimicrobial agent administration can decrease the risk of later cardiac complications, risk factors for pediatric *Streptococcus* infection, including environmental exposure, remain poorly understood. Therefore, investigating factors associated with pediatric *Streptococcus* infection is crucial for its prevention and early treatment. Accordingly, identification of both risk factors and potential preventative factors for pediatric *Streptococcus* infection therefore remains a pressing public health issue. However, the potential impacts of environmental factors, including metal and trace element exposure, on pediatric *Streptococcus* infection remain poorly understood.

Both toxic metals and essential trace elements, such as selenium (Se) and manganese (Mn), have the potential to influence key biological processes even in very small amounts. Among the essential trace elements, selenium has garnered attention for its influence on immune function, where it has been reported to improve fetal immune system development and regular immune system maintenance among children [3,4]. Thus, selenium may be expected to be associated with reduced risk of pediatric *Streptococcus* infection. In addition, selenium can mitigate mercury toxicity at multiple stages of mercury metabolism, including its transport, bioavailability, and toxicological consequences, though the mechanism of interaction between selenium and mercury is not yet fully understood [5].

Despite the potential for essential trace elements and toxic metals to influence immune function, to the best of our knowledge, there are no reports of a large-scale epidemiological study of pediatric *Streptococcus* infection and prenatal metal and trace element exposure. The present study implemented a prospective birth cohort to evaluate blood metal and trace element concentrations during pregnancy and the risk of developing pediatric *Streptococcus* infection during a one-year period, measured at age three to four years.

## 2. Methods

### 2.1. Study design and population

**Fig 1:**
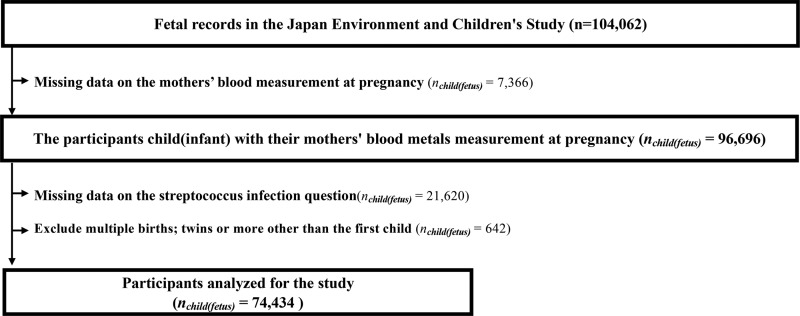
Study participant inclusion flow chart.

The present study used data from the Japan Environment and Children’s Study (JECS), which is an ongoing prospective birth cohort study. The JECS recruited pregnant mothers from 15 Regional Centres across Japan from 2011 to 2014, including more than 100,000 pregnancies (jecs-ta-20190930 and jecs-qa-20210401 datasets). The precise methodology of the JECS is presented in Kawamoto et al. [6]. During the recruitment period, the JECS covered roughly 45% of total live births within the Study Area [6,7]. The present study included participants for whom maternal metal and trace element levels were measured during a pregnancy which resulted in the mother’s first single live birth. We excluded anyone who did not answer the questionnaire item related to *Streptococcus* infection, giving a total of 74,434 research participants. Our study flow chart is presented in Fig 1.

### 2.2. Assessment of maternal metal and trace element exposure levels

The present study collected blood samples from mothers who were participating in the JECS study during the second (gestational age: 14–27 weeks) or third trimester ( ≥ 28 weeks) [7]. Approximately half of the blood samples were collected during the second and third trimesters, respectively [7]. The methodology employed for the measurement of metals and trace elements is described in Nakayama et al. [8]. In brief, the JECS collected maternal blood samples in order to measure their cadmium (Cd), lead (Pb), mercury (Hg), manganese (Mn), and selenium (Se) levels. Blood samples were collected by medical staff during the participant’s visit to the cooperating health care provider. The blood samples for chemical analysis were collected in sodium ethylenediaminetetraacetic acid (EDTA), and were transferred to a central laboratory, aliquoted into cryo-biobank tubes, and stored at -80°C until analysis within 48 hours after blood sampling via land or air transportation [7,8].

### 2.3. Assessment of *Streptococcus* infection incidence

The primary outcome was the incidence of pediatric *Streptococcus* infection during the past one-year period, as reported on questionnaire survey completed by parents conducted when the children were aged between three and four years. Occurrence of pediatric *Streptococcus* infection was defined as a report of pediatric *Streptococcus* infection diagnosed by a physician in the past year when surveyed at age three to four years (S1 Text). The outcome of “*Streptococcus* infection” was deemed to primarily imply GAS pharyngitis [9].

### 2.4. Statistical analysis

We performed descriptive statistics to assess the basic characteristics of mothers and their children. We then implemented univariable and multivariable logistic regressions using incidence of pediatric *Streptococcus* infection as the outcome variable. The main independent variables were maternal metal and trace element levels. We conducted the multivariable regressions adjusting for covariates selected based on a directed acyclic graph (DAG). When conducting regressions, the metal and trace element levels were normalized using log 2 transformation because their distributions were positively skewed [10]. When conducting uni- and multi-variable regressions, we implemented a False Discovery Rate (FDR) correction method using the Benjamini-Hochberg procedure in order to adjust for multiple comparisons [11]. P-values under 0.05 were defined as statistically significant. The statistical analyses were performed using R software (version 4.0.3; R Foundation for Statistical Computing, Vienna, Austria). We conducted Quantile g-computation to investigate the mixture effects of the metals and trace elements (Cd, Pb, Hg, Mn, and Se) on GAS infection, adjusting for covariates. We implemented the Quantile g-computation using the R package “qgcomp” [12].

### 2.5. Covariates

**Fig 2:**
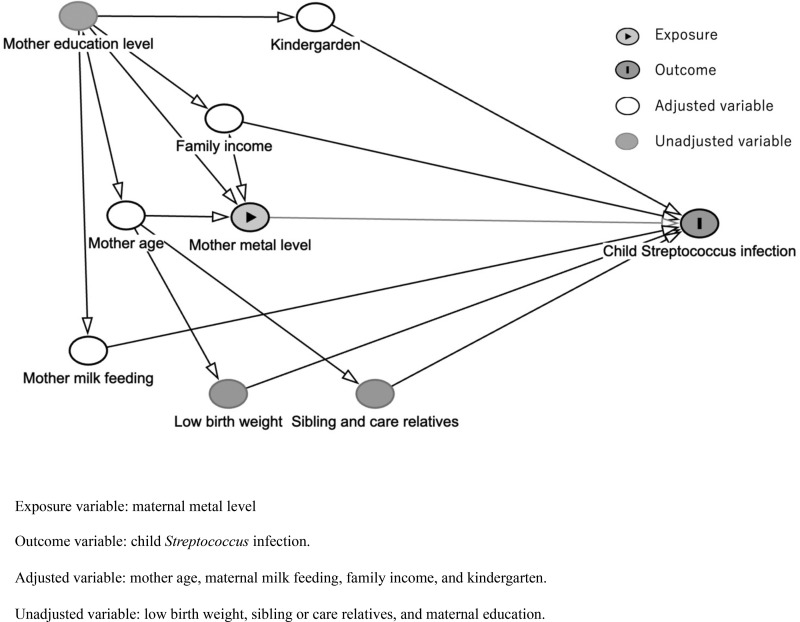
Directed acyclic graph in the present study.

We chose covariates based on clinical importance using our DAG, which was generated with the R package “Dagitty” [13]. (Fig 2) We identified the following potential covariates: maternal age at delivery, maternal milk feeding at six months after delivery, family income at the second or third trimester, child kindergarten attendance at age four years. As for family income, statistics from the 2019 Comprehensive Survey of Living Conditions published by the Ministry of Health, Labour and Welfare in Japan show that the average income per household is in the region of 4 million yen at the median. Based on this figure, we divided family income into two strata for analysis: low income (annual income <  4 million yen) and moderate or higher income (annual income ≥  4 million yen).

### 2.6. Additional analysis

When statistical significance was observed in the regression analysis for one or more of the five target metals and trace elements, we conducted an additional quartile regression analysis, dividing the data for the explanatory variable into four intervals (Q1: values below the first quartile; Q2: values below the median and above the first quartile; Q3: values below the third quartile and above the median; Q4: values above the third quartile), and setting Q1 as the reference variable. Finally, we conducted regression analysis with selenium, mercury, and their interaction term adjusted for covariates in order to investigate the potential additive interaction between selenium and mercury on pediatric *Streptococcus* infection incidence [14].

### 2.7. Sensitivity analysis

As a sensitivity analysis, we conducted univariable and multivariable logistic regression with data from the same children during the one-year period from child age two to three, instead of age three to four as in the main analysis. We expected that the same trend would be observed across different outcome durations if metal or trace element exposure affects the risk of pediatric *Streptococcus* infection.

#### Ethics approval and consent to participate.

The JECS protocol was reviewed and approved by the Ministry of the Environment’s Institutional Review Board on Epidemiological Studies (No. 100910001), and the Ethics Committees of all participating institutions. The JECS obtained written informed consent from all study participants. Moreover, the JECS followed the ethical principles of the Declaration of Helsinki and its revisions. Written informed consent for participation in the study were obtained from individual mothers and their partners, and for children from their parent or guardian [15].

## 3. Results

The basic characteristics of the participants in the present study are shown in Table 1. Among the 74,434 children, about 8.1% (n =  6,021) experienced pediatric *Streptococcus* infections during the past one-year as reported at age three to four. There were no obvious differences in maternal age between the pediatric *Streptococcus* and non- pediatric *Streptococcus* groups. There was a higher proportion of male children in the pediatric *Streptococcus* group. Moreover, the children in the pediatric *Streptococcus* group had higher rates of maternal milk feeding during the first month after birth, low birth weight, presence of siblings or other family members in their homes, and kindergarten attendance. The distributions of the metal and trace element levels are summarized in Table 2. For selenium, the mean and median levels were 170 and 168 ng/g, respectively, with minimum and maximum ranging from 83 to 976 ng/g.

**Table 1:** Study participant characteristics.

	Streptococcus group	Non-Streptococcus group	Overall
	(N = 6021)	(N = 68413)	(N = 74434)
**Mother age at delivery, years**			
Mean (SD)	31.5 (4.79)	31.5 (4.90)	31.5 (4.89)
Median [Q1, Q3]	31.0 [28.0, 35.0]	31.0 [28.0, 35.0]	31.0 [28.0, 35.0]
**Maternal milk intake within 1 month of birth**			
Positive	3266 (54.2%)	36820 (53.8%)	40086 (53.9%)
Negative	2719 (45.2%)	31318 (45.8%)	34037 (45.7%)
Missing	36 (0.6%)	275 (0.4%)	311 (0.4%)
**Child sex**			
Male	3360 (55.8%)	34760 (50.8%)	38120 (51.2%)
Female	2661 (44.2%)	33653 (49.2%)	36314 (48.8%)
**Child weight less than 2500 g at delivery**			
Positive	526 (8.7%)	5573 (8.1%)	6099 (8.2%)
Negative	5478 (91.0%)	62708 (91.7%)	68186 (91.6%)
Missing	17 (0.3%)	132 (0.2%)	149 (0.2%)
**Presence of sibling or other relative in home**			
Positive	1601 (26.6%)	17234 (25.2%)	18835 (25.3%)
Negative	4420 (73.4%)	51179 (74.8%)	55599 (74.7%)
**Kindergarten attendance**			
Positive	5481 (91.0%)	59412 (86.8%)	64893 (87.2%)
Negative	142 (2.4%)	4733 (6.9%)	4875 (6.5%)
Missing	398 (6.6%)	4268 (6.2%)	4666 (6.3%)
**Family income**			
Moderate- or higher-income family	3479 (57.8%)	39139 (57.2%)	42618 (57.3%)
Low-income family	2147 (35.7%)	24686 (36.1%)	26833 (36.0%)
Missing	395 (6.6%)	4588 (6.7%)	4983 (6.7%)

**Table 2:** Metal and trace element concentrations in maternal blood (n =  74,434).

Metal and trace element	Mean	Standard deviation	Median	Minimum	5%	25%	75%	95%	Maximum
Manganese, ng/g (Mn)	15.9	4.7	15.3	3.9	9.5	12.6	18.6	24.4	60.8
Selenium, ng/g, (Se)	170	20	168	83	141	156	181	205	976
Lead, ng/g (Pb)	6.3	2.8	5.8	1.2	3.4	4.7	7.3	10.6	103.0
Cadmium, ng/g (Cd)	0.7	0.4	0.7	0.1	0.3	0.5	0.9	1.4	5.2
Mercury, ng/g (Hg)	4.2	2.5	3.7	0.3	1.5	2.6	5.2	8.8	43.7

Outcomes of univariable and multivariable regressions for pediatric *Streptococcus* incidence are presented in Table 3. The univariable regression of selenium levels provided an odds ratio of 0.78; 95% confidence interval (CI) [0.66-0.91] and mercury provided an odds ratio of 0.95; 95% CI [0.92-0.98]. The 95% CI odds ratios for the other metals and trace element all crossed 1. Multivariable regression analysis adjusted for maternal age, maternal milk feeding, family income, and child attendance at kindergarten provided an odds ratio of 0.80; 95% CI [0.68-0.95] for selenium, and 0.95; 95% CI [0.91-0.98] for mercury. Quantile-g-computation regression provided an odds ratio of 0.81, 95% CI [0.67-0.97], *p* =  0.02. Our quantile g-computation also shows that selenium had heavy negative weight while cadmium had heavy positive weight. And the others such as magnesium, lead and mercury had little weight. (S1 Fig), which provided that over 69% of negative weight was Selenium, while cadmium has positive weight on pediatric *Streptococcus* infection. Our sensitivity analysis found the same trend as our primary regression analysis (S1 Table).

**Table 3:** Univariable and multivariable regression results.

Univariable analysis	Odds ratio	lower 95% CI*	Upper 95% CI*	P-value	Q-value ^†^
Manganese (Mn) ^‡^	0.99	0.93	1.05	0.67	0.84
Selenium (Se) ^‡^	0.78	0.66	0.91	0.002	0.0075
Lead (Pb)^‡^	0.97	0.92	1.02	0.21	0.35
Cadmium (Cd)^‡^	1.00	0.96	1.04	0.95	0.95
Mercury (Hg)^‡^	0.95	0.92	0.98	0.003	0.0075
Multivariable analysis	Odds ratio	lower 95%CI*	Upper 95% CI*	P-value	Q-value ^†^
Manganese (Mn)**^,‡^	0.99	0.93	1.06	0.72	0.72
Selenium (Se)**^,‡^	0.80	0.68	0.95	0.009	0.0225
Lead (Pb)**^,‡^	0.98	0.93	1.03	0.43	0.71
Cadmium (Cd)**^,‡^	1.01	0.97	1.06	0.57	0.71
Mercury (Hg)**^,‡^	0.95	0.91	0.98	0.002	0.01

*CI; Confidence interval.

**Multivariable regressions were adjusted for maternal age, maternal milk feeding, family income, and child attendance at kindergarten.

†False discovery rate (FDR) correction with the Benjamini-Hochberg procedure produced q-values.

‡Exposure to each metal and trace element was calculated individually. Log 2 transformed, ng/dl.

We conducted additional regression analysis on selenium exposure. The quartile regression results for selenium are shown in Table 4. The regressions in Table 4 show that selenium levels in the Q4 range had the lowest odds ratios. Our interaction analysis did not support a finding of interaction between selenium and mercury exposure (S2 Table).

**Table 4:** Quartile regression results for Selenium level.

Univariable^†^	Odds ratio	Lower 95% CI*	Upper 95% CI*	P-value
Group Q1	Reference	–	–	–
Group Q2	0.97	0.9	1.04	0.34
Group Q3	0.96	0.89	1.04	0.3
Group Q4	0.89	0.82	0.95	0.002
**Multivariable^†^**	**Odds ratio**	Lower 95% CI*	Upper 95% CI*	**P-value**
Group Q1**	Reference	–	–	–
Group Q2**	0.94	0.87	1.02	0.15
Group Q3**	0.97	0.90	1.05	0.49
Group Q4**	0.88	0.81	0.96	0.0024
**Univariable**	**Odds ratio**	Lower 95% CI*	Upper 95% CI*	**P-value**
Group Q1	1.04	0.96	1.11	0.34
Group Q2	Reference	–	–	–
Group Q3	1.00	0.92	1.07	0.92
Group Q4	0.92	0.85	0.99	0.03
**Multivariable**	**Odds ratio**	Lower 95% CI*	Upper 95% CI*	**P-value**
Group Q1**	1.06	0.98	1.15	0.15
Group Q2**	Reference	–	–	–
Group Q3**	1.03	0.95	1.12	0.46
Group Q4**	0.94	0.86	1.01	0.11
**Univariable**	**Odds ratio**	Lower 95% CI*	Upper 95% CI*	**P-value**
Group Q1	1.04	0.97	1.12	0.30
Group Q2	1.00	0.93	1.08	0.92
Group Q3	Reference	–	–	–
Group Q4	0.92	0.85	0.99	0.04
**Multivariable**	**Odds ratio**	Lower 95% CI*	Upper 95% CI*	**P-value**
Group Q1**	1.03	0.95	1.11	0.49
Group Q2**	0.97	0.90	1.05	0.46
Group Q3**	Reference	–	–	–
Group Q4**	0.91	0.84	0.99	0.020

^Q^1–Q4, intervals 1 through 4.

*CI; Confidence interval.

**Multivariable regressions were adjusted for maternal age, maternal milk feeding, family income, and child attendance at kindergarten.

†Q1-4 were set as ordinal variables.

## 4. Discussion

Our findings do not indicate a significant correlation between maternal cadmium, lead, or manganese levels and later pediatric *Streptococcus* infection among children. However, maternal selenium and mercury levels were found to have statistically significant inverse associations with pediatric *Streptococcus* infection. Furthermore, the negative correlations of selenium found on Quantile g-computation analysis are consistent with the results of the regression analysis. Moreover, the additional quartile regression analyses revealed an inverse association between high maternal serum selenium levels and later pediatric *Streptococcus* infection among their children. Finally, sensitivity analyses conducted using data collected over different observation periods showed the same trend as the primary regression analyses, supporting our main findings.

The present study suggests that there is an inverse association between higher selenium levels and pediatric *Streptococcus* infection. This observed association should be considered both in terms of selenium’s immunological and pediatric *Streptococcus* -specific effects. Immunologically, selenium is gathering attention based on reports of its capacity to confer multiple physiological health benefits. Firstly, selenium is an indispensable trace mineral [16]. Selenium influences the immune response to bacterial infection through a number of mechanisms, involving immune cell activation, [17] inflammasome activation, [18] antioxidant effects, [19] and selenoproteins [20,21]. In particular, selenoproteins have been reported to have a wide range of multifaceted actions, from antioxidant to anti-inflammatory effects [22]. Accordingly, low selenium levels are associated with increased mortality, impaired immune function, and cognitive decline. Conversely, higher selenium levels or selenium supplementation have been reported to have antiviral effects [22].

Next, selenium plays an important role in fetal immune system development. In a rat study investigating the effects of dietary selenium intake on neonatal immune cell differentiation, Dylewski et al. reported that low maternal selenium intake was associated with low plasma selenium among their pups, and that pups receiving low-selenium milk showed statistically significant impairment of thymocyte activation, as well as decreased CD8 cytotoxic T cells, CD 2 T cells, and panB cells among pups nursed by mothers receiving low-selenium diets.^3^ Their study implies that maternal selenium intake impacts neonatal selenium levels, which in turn affects neonatal immune system development.

There are also reports focusing on selenium’s *Streptococcus*-specific effects. Selenium enrichment of yeasts and lactic acid bacteria (LAB) has recently appeared as a novel concept [23]. Shahmoradi et al. investigated the application of selenium nanoparticles for enhancing the efficacy of photodynamic inactivation of planktonic communities and the biofilm of *Streptococcus mutans* on tooth surfaces [24]. Their study shows that photodynamic treatment with selenium nanoparticles had a high potential to remove *Streptococcus mutans* biofilms. Hence, selenium may be expected to help reduce *Streptococcus* infection risk among children.

In particular, the results of our quantile univariable and multivariable regressions show that selenium levels in the Q4 high dose range had lower odds ratios compared with the other ranges (Q1 – Q3), other than the reference Q2 on the multivariable regression. These results suggest that higher maternal selenium levels may reduce the risk for *Streptococcus* infection among children. These results are in accordance with previous reports which suggest that selenium can have immune enhancement effects against infections [3,22–24]. If future epidemiological reports indicate that children have poor selenium intake, infection prevention strategies through selenium supplementation might be considered as a possible public health intervention.

Importantly, both deficiency and excessive selenium intake can be harmful to humans [25]. Selenium toxicity can arise with both acute and chronic over-exposure [26]. Various pediatric epidemiological studies have investigated the potential risks and benefits of maternal selenium intake, though not in the context of infectious diseases. Everson et al., in a hospital-based birth cohort study in the United States, reported that selenium exposure decreased the odds of intrauterine growth restriction. In a prospective birth cohort study conducted in France, Baïz et al. reported that prenatal selenium exposure may protect against wheezing in children by the age of 3 years old [27]. Further, Solé-Navais et al. reported that a selenium-rich diet for the mother during pregnancy may be beneficial to fetal growth, based on data from a Norwegian birth cohort [28]. Conversely, Lee et al., in a US-based birth cohort study, reported that elevated prenatal selenium exposure increased the risk of neurodevelopmental disorders among children [29]. Therefore, the results of the present study do not imply that increased selenium intake would be unambiguously beneficial. Even provided that higher maternal serum selenium levels were associated with reduced rates of pediatric *Streptococcus* infection in this study, selenium intake should be determined with consideration for the risk profiles of diseases other than pediatric *Streptococcus* infection. Further studies are therefore warranted to investigate the appropriate range of maternal serum selenium levels.

Our statistical results also show that maternal mercury exposure may have an inverse association with pediatric *Streptococcus* infection. However, mercury exposure is unlikely to suppress pediatric *Streptococcus* infection directly. Firstly, our quantile -g-computation show little weight on *Streptococcus* infection as the mixture effect of the metals and trace elements. Secondly, unlike selenium, mercury is a metal, and not an essential trace element. Next, as the World Health Organization (WHO) has reported, mercury exposure can threaten both fetal and child development [30]. Thus, mercury would not be expected contribute to immune reinforcement among children. In the present study, it is possible that mercury exposure could be a proxy for fish intake and that elevated mercury levels were not associated with adverse events among fish eaters [31]. Increased fish intake is reportedly correlated with high health competency among parents [32]. Some reports suggesting that selenium may play an antagonistic role in mercury exposure. Thus, the present study investigated the additive interaction function of selenium on mercury, but no significant interaction was observed.

The DAG presented in Fig 2 was used in the covariate selection process. DAGs are gathering attention as a means of selecting confounders as covariates in order to reduce bias [33–35]. We presumed that maternal milk feeding may affect immunity against infectious diseases among children [36]. We also presumed that maternal age at pregnancy was associated with maternal blood selenium levels [37–41]. Kindergarten attendance was included because of its expected relationship with pathogen exposure. Furthermore, socio-economic status is regarded an important factor when assessing epidemiological associations, including infectious disease risk [42]. Family income and maternal education level are often used to estimate socio-economic status [43]. Accordingly, given the association in our DAG, we adjusted for the following covariates: maternal age at delivery, maternal milk feeding, kindergarten attendance, and family income.

There are some limitations in the present study. First, pediatric *Streptococcus* infection was confirmed using a patent-completed questionnaire-based method which raises some concerns. In Japan, GAS pharyngitis is often referred to using the abbreviated expression “*yourenkin kansenshou*” [44], which has been translated as “*Streptococcus* infection” in medical reports [45,46]. As used by members of the general public and in some medical practice and public health settings, y*ourenkin kansenshou* is considered to be equivalent to “strep throat” [47,48] in English. Thus, we presumed that the outcome “*Streptococcus* infection” on the questionnaire used in this study (S1 Text) refers to GAS pharyngitis. However, “*Streptococcus* infection” can also include pharyngitis caused by other streptococci, such as those belonging to Group B [49]. Moreover, in a broader sense, “*Streptococcus* infection” could refer to various other GAS-related conditions, such as otitis media, cellulitis, glomerulonephritis, and toxic shock syndrome [50]. Therefore, we must acknowledge the possibility that some of the responses to the questionnaire item on “*Streptococcus* infection” referred to GAS-related conditions other than pharyngitis or to diseases caused by other streptococci. While a questionnaire item using a more specific disease name would be more precise, the term “*Streptococcus* infection” was selected because it was deemed more understandable for the non-healthcare professionals being asked to complete the questionnaire. In addition, one of the limitations of a questionnaire-based survey is recall bias. However, because children are suspended from attendance at kindergarten or daycare, parents may be more likely to recall the event, which may reduce the risk of recall bias. In addition, JECS asked the parents about diagnoses made by physicians, which may be easier to recall. Next, we did not receive responses for more than a quarter of the children in the JECS cohort and thus excluded them from our analyses, which raises the possibility of selection bias. Furthermore, while maternal levels of metals and trace elements were measured, exposure to metals and trace elements among their fetuses and children was not measured directly. Finally, the JECS study did not measure other microelements such as iron and zinc. These metals are also indispensable for child growth. Further studies were warranted to investigate the potential effects of these metals on bacterial infections.

## 5. Conclusions

The present study implies that higher maternal selenium levels have an inverse association with later pediatric *Streptococcus* incidence among their children. Further research is warranted to investigate the association between pediatric selenium function and immune responses to infectious diseases, with particular focus on streptococcal infections.

## Supporting Information

S1 Table Sensitivity analysis with univariable and multivariable regression results. (DOCX)

S2 Table Interaction term regression results. (DOCX)

S1 Fig Quantile-g-computation results. (DOCX)

S1 Text English translation of streptococcal infection questionnaire item. (DOCX)

## Abbreviations

Cd: cadmium
CI: confidence interval
EDTA: ethylenediaminetetraacetic acid
FDR: false discovery rate
GAS: group A β-hemolytic *Streptococcus pyogenes*
Hg: Mercury
JECS: Japan Environment and Children’s Study
LAB: lactic acid bacteria
Mn: Manganese
Pb: Lead
Q1: First Interval
Q2: Second Interval
Q3: Third Interval
Q4: Fourth Interval
Se: Selenium
WHO: World Health Organization.
