# Supplementary material for: Association between prenatal exposure to maternal metal and trace elements and Streptococcus infection: A prospective birth cohort in the Japan Environment and Children’s Study
Source: PLoS One. 2025 Feb 27;20(2):e0319356. doi: 10.1371/journal.pone.0319356 (PMC11867319; doi:10.1371/journal.pone.0319356)
Supplement: S1 Table — (DOCX) [file pone.0319356.s001.docx]

Supplementary Table 1. Sensitivity analysis with univariable and multivariable regression results.

| Univariable analysis | Odds ratio | lower 95% CI* | Upper 95% CI* | P-value | Q-value ^†^ |
| --- | --- | --- | --- | --- | --- |
| Manganese (Mn) ^‡^ | 1.02 | 0.95 | 1.10 | 0.60 | 0.75 |
| Selenium (Se) ^‡^ | 0.69 | 0.57 | 0.83 | 0.0001 | 0.0005 |
| Mercury (Hg) ^‡^ | 0.98 | 0.92 | 1.04 | 0.45 | 0.75 |
| Cadmium (Cd) ^‡^ | 1.00 | 0.95 | 1.05 | 0.97 | 0.98 |
| Lead (Pb) ^‡^ | 0.94 | 0.91 | 0.98 | 0.005 | 0.0125 |
|  |  |  |  |  |  |
| Sensitivity analysis | Odds ratio | lower 95% CI* | Upper 95% CI* | P-value | Q-value ^†^ |
| Manganese (Mn)^** ‡^ | 0.99 | 0.91 | 1.07 | 0.73 | 0.74 |
| Selenium (Se)^** ‡^ | 0.71 | 0.58 | 0.88 | 0.0016 | 0.004 |
| Mercury (Hg)^** ‡^ | 0.96 | 0.90 | 1.03 | 0.26 | 0.43 |
| Cadmium (Cd)^** ‡^ | 0.99 | 0.94 | 1.05 | 0.74 | 0.74 |
| Lead (Pb)^** ‡^ | 0.92 | 0.88 | 0.96 | 0.0004 | 0.002 |

*CI; Confidence interval.

**Multivariable regressions were adjusted for maternal age, maternal milk feeding, family income, and child attendance at kindergarten.

† False discovery rate (FDR) correction with the Benjamini-Hochberg procedure produced q-values.

‡Exposure to each metal and trace element was calculated individually. Log 2 transformed, ng/dl.
