# Supplementary material for: Association between prenatal exposure to maternal metal and trace elements and Streptococcus infection: A prospective birth cohort in the Japan Environment and Children’s Study
Source: PLoS One. 2025 Feb 27;20(2):e0319356. doi: 10.1371/journal.pone.0319356 (PMC11867319; doi:10.1371/journal.pone.0319356)
Supplement: S2 Table — (DOCX) [file pone.0319356.s002.docx]

Supplementary Table 2. Interaction term regression results.

| Univariable analysis | Odds ratio | lower 95%CI* | Upper 95% CI* | P-value |
| --- | --- | --- | --- | --- |
| Selenium (Se) ^‡^ | 0.73 | 0.49 | 1.08 | 0.11 |
| Mercury (Hg) ^‡^ | 0.52 | 0.13 | 2.17 | 0.36 |
| Interaction with Se and Hg^‡^ | 1.09 | 0.89 | 1.31 | 0.40 |

*CI; Confidence interval.

**Multivariable regressions were adjusted for maternal age, maternal milk feeding, family income, and child attendance of kindergarten.

‡Log 2 transformed, ng/dl.
