# Supplementary material for: Association between prenatal exposure to maternal metal and trace elements and Streptococcus infection: A prospective birth cohort in the Japan Environment and Children’s Study
Source: PLoS One. 2025 Feb 27;20(2):e0319356. doi: 10.1371/journal.pone.0319356 (PMC11867319; doi:10.1371/journal.pone.0319356)
Supplement: S1 Fig — (DOCX) [file pone.0319356.s003.docx]

Supplementary Figure 1. Quantile-g-computation results.


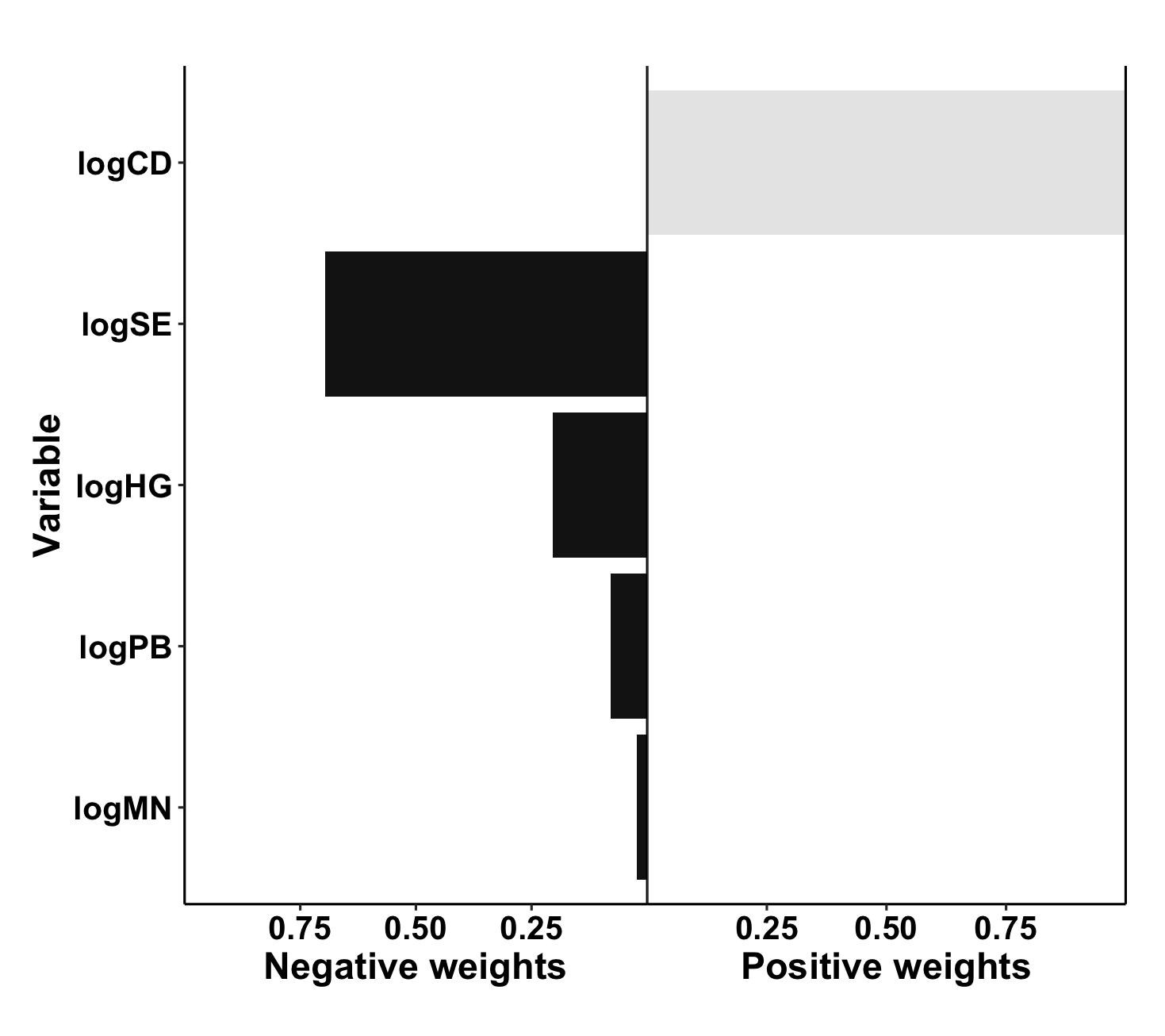


CD, Cadmium, Se; Selenium, Hg; Mercury, Pb; Lead, Mn; Manganese.

Quantile g computation was adjusted for maternal age, maternal milk feeding, family income, and child attendance at kindergarten.
