# Supplementary material for: Association between prenatal exposure to maternal metal and trace elements and Streptococcus infection: A prospective birth cohort in the Japan Environment and Children’s Study
Source: PLoS One. 2025 Feb 27;20(2):e0319356. doi: 10.1371/journal.pone.0319356 (PMC11867319; doi:10.1371/journal.pone.0319356)
Supplement: S1 Text — (DOCX) [file pone.0319356.s004.docx]

Supplementary text 1. English translation of streptococcal infection questionnaire item.

Has your child been diagnosed by a doctor with any of the following diseases from age three to the present (age four) ?

This includes cases where the child is still continuing to visit the doctor or receive treatment.

- Streptococcal infection　(Check box)

Results

|  | Total | Strep group* | Non-strep group** | Unknown | Strep/Non-strep proportion (%) |
| --- | --- | --- | --- | --- | --- |
| All JECS participants | 104062 | 6321 | 71816 | 25925 | 8.802 |
| Participants with blood metal and trace element measurement | 96696 | 6071 | 69005 | 21620 | 8.798 |
| Participants in the present study | 74434 | 6021 | 68413 | 0 | 8.801 |

*Strep group: Streptococcal infection positive group (age three to four).

**Non-strep group: Streptococcal infection negative group (age three to four).
